# Supplementary material for: Rapid turnover of life-cycle-related genes in the brown algae
Source: Genome Biol. 2019 Feb 14;20:35. doi: 10.1186/s13059-019-1630-6 (PMC6374913; doi:10.1186/s13059-019-1630-6)
Supplement: Supplementary file 3 — Protein alignments, corresponding Gblocks html file and CDS sequences for the lineage conserved gametophyte-biased genes. (ZIP 2109 kb) [file 13059_2019_1630_MOESM3_ESM.zip › Con_GA_alignments/Ec-28_001410.aa_ali.fasta-gb.htm]

Ec-28\_001410.aa.fasta.trans.aa\_ali.fasta


## Gblocks 0.91b Results

Processed file: **Ec-28\_001410.aa.fasta.trans.aa\_ali.fasta**  
Number of sequences: **2**  
Alignment assumed to be: **Protein**  
New number of positions: **447** (selected positions are underlined in blue)

```
                         10        20        30        40        50        60
                 =========+=========+=========+=========+=========+=========+
Ec-28_001410     MPLAFKFSAVASLNAALQLLVLLRCTSITVVDAFVALHPSAAVSEIVWSRHR-TDTLRTS
MSTRG.50701.1    MHHAVKFSVAGSCNAAL-LVALLSCITGQSVDAFAIIRSIAAISESTAGKCRRCAALSAT
                 ################# ##################################        


                         70        80        90       100       110       120
                 =========+=========+=========+=========+=========+=========+
Ec-28_001410     ATPPPLSPRPSRSHMTSLPKRIRPVPLPEAVPATPVPRTRCHAMHGNNYAAHDDDRAGGR
MSTRG.50701.1    WSSASTTARPARSRWIAP--SFTPVPLSCGRRRCKAGYGGCHATR----CCVDDDPLKWA
                                                                             


                        130       140       150       160       170       180
                 =========+=========+=========+=========+=========+=========+
Ec-28_001410     ADIHNLDAPGQQERHHFGGAPAGIGDDVAASRDIFG-EQGGGFGWLREFGNGKTDIAGLV
MSTRG.50701.1    ASFES-AAPDQRRSNNYGCPPAAVQHSAASRRDVSDAGEASGFEWLREFGNGKMDVAGLV
                        ##########################        ###################


                        190       200       210       220       230       240
                 =========+=========+=========+=========+=========+=========+
Ec-28_001410     LSTVLLMTAVGAPLGAAADLGVVDGRVLDPS----------SLSHGPTAVRLNVENAVPV
MSTRG.50701.1    MSTVFLMTAV-APMGIAADLGVVDGGRLDQHPPRQRLVGHLTVEHPGRVGPLLGEAKATP
                 ########## ##################               ###########     


                        250       260       270       280       290       300
                 =========+=========+=========+=========+=========+=========+
Ec-28_001410     PTAEKARANRVGALNLGGLPPRRPPVTRVAGVESEVGAVGEASTRRTGPLAKMAGDSMSA
MSTRG.50701.1    AGLATSRTERVGALRLGDMASRHAPVPPSSGVNSRGTTDSETIPRRTGPLAQMAGNAASS
                       ######################################################


                        310       320       330       340       350       360
                 =========+=========+=========+=========+=========+=========+
Ec-28_001410     VRDIAHRAGAIAASRGPGRESAPGAPGPLRTAKPDVHKEPPPEKTEDSGSVVGDDHPHTS
MSTRG.50701.1    ARGVAERAMAIRS-RAAEQDRMSDSSRSTDAANLVVREEKPRAKTRISP-----------
                 ###########                    #################            


                        370       380       390       400       410       420
                 =========+=========+=========+=========+=========+=========+
Ec-28_001410     LQRGVPSAGRSTTVNAVRSGGQVEDGTGAAAKAARSDQLEELENEHTKLFGDSLPLSTLP
MSTRG.50701.1    ----LFSNGGVNTGLALSPGRRAATKT--AVQPDPAELLNRLEVGHVELYGESLPLPTTP
                       #####################  ###############################


                        430       440       450       460       470       480
                 =========+=========+=========+=========+=========+=========+
Ec-28_001410     DSFNRAIRRALPSVLPPAMAVFLTVSMPAVILLLKGVGTTLVAVAKGYGKADRRRSDDGH
MSTRG.50701.1    ESFNRAIRKALPALVPPAMAIFLTVSMPAVVLLLKGIGTTVVAVAKGYGKGDRRRAEDEF
                 ############################################################


                        490       500       510       520       530       540
                 =========+=========+=========+=========+=========+=========+
Ec-28_001410     DVGSALEVGFGGGGGVFGGSVGGEGKRRERGPETPGSAFQMFLGGNGGSGGVDAGGSGHG
MSTRG.50701.1    EAGAPLQVGFGGDG-------SGRDRLRGRSAENPGPAFQMFLGGNGGDGGVNRDRPSEL
                 ##############        ##############################        


                        550       560       570       580       590       600
                 =========+=========+=========+=========+=========+=========+
Ec-28_001410     GDVDTRAGGPMGDSSGGVAPFAPPLTVQQVSTERQGFFSKPRQQHP-------QLKDTGG
MSTRG.50701.1    DIAERKSSLPADRSSMEETRSYSSLPPQQVETDRQGFFSIKHNRSNGGGSGGGGKNPSGG
                                         ###############                   ##


                        610       620       630       640       650       660
                 =========+=========+=========+=========+=========+=========+
Ec-28_001410     GFAVGDDAMAAMAMAAMTQELTVMKHSLQKEVESMRNELERASDDSRRLTDKLCRLEEDN
MSTRG.50701.1    GFAVGDDAMTAMAMVAMTQELNTMKQSLQTEVETMKQELERASDDSRRLADKLIRLEX--
                 #########################################################   


                        670       680       690       700
                 =========+=========+=========+=========+======
Ec-28_001410     RRLEAVVSRLGGQDARRLKAQLFSLLASPSMGQLGQGNPSTQLSPA
MSTRG.50701.1    ----------------------------------------------
```

```
Parameters used
Minimum Number Of Sequences For A Conserved Position: 2
Minimum Number Of Sequences For A Flanking Position: 2
Maximum Number Of Contiguous Nonconserved Positions: 8
Minimum Length Of A Block: 10
Allowed Gap Positions: None
Use Similarity Matrices: Yes
```

```
Flank positions of the 13 selected block(s)
Flanks: [1  17]  [19  52]  [128  153]  [162  190]  [192  209]  [225  235]  [247  311]  [332  348]  [367  387]  [390  494]  [503  532]  [565  579]  [599  657]  

New number of positions in Ec-28_001410.aa.fasta.trans.aa_ali.fasta-gb:  447  (63% of the original 706 positions)
```
